# The added value of PSMA PET/MR radiomics for prostate cancer staging

**DOI:** 10.1007/s00259-021-05430-z

**Published:** 2021-07-13

**Authors:** Esteban Lucas Solari, Andrei Gafita, Sylvia Schachoff, Borjana Bogdanović, Alberto Villagrán Asiares, Thomas Amiel, Wang Hui, Isabel Rauscher, Dimitris Visvikis, Tobias Maurer, Kristina Schwamborn, Mona Mustafa, Wolfgang Weber, Nassir Navab, Matthias Eiber, Mathieu Hatt, Stephan G. Nekolla

**Affiliations:** 1 School of Medicine, Department of Nuclear Medicine, Klinikum rechts der Isar, Technical University Munich, Munich, Germany; 2 School of Medicine, Department of Urology, Klinikum rechts der Isar, Technical University Munich, Munich, Germany; 3 INSERM, UMR 1101, LaTIM, Univ Brest, Brest, France; 4 Department of Urology and Martini-Klinik Prostate Cancer Center, University Hospital Hamburg-Eppendorf, Hamburg, Germany; 5 School of Medicine, Institute of Pathology, Klinikum rechts der Isar, Technical University Munich, Munich, Germany; 6 School of Computer Science, Computer Aided Medical Procedures and Augmented Reality, Technical University Munich, Munich, Germany

**Keywords:** Prostate cancer, PET/MRI, PSMA, Radiomics, Gleason score

## Abstract

**Purpose:**

To evaluate the performance of combined PET and multiparametric MRI (mpMRI) radiomics for the group-wise prediction of postsurgical Gleason scores (psGSs) in primary prostate cancer (PCa) patients.

**Methods:**

Patients with PCa, who underwent [^68^ Ga]Ga-PSMA-11 PET/MRI followed by radical prostatectomy, were included in this retrospective analysis (n = 101). Patients were grouped by psGS in three categories: ISUP grades 1–3, ISUP grade 4, and ISUP grade 5. mpMRI images included T1-weighted, T2-weighted, and apparent diffusion coefficient (ADC) map. Whole-prostate segmentations were performed on each modality, and image biomarker standardization initiative (IBSI)-compliant radiomic features were extracted. Nine support vector machine (SVM) models were trained: four single-modality radiomic models (PET, T1w, T2w, ADC); three PET + MRI double-modality models (PET + T1w, PET + T2w, PET + ADC), and two baseline models (one with patient data, one image-based) for comparison. A sixfold stratified cross-validation was performed, and balanced accuracies (bAcc) of the predictions of the best-performing models were reported and compared through Student’s t-tests. The predictions of the best-performing model were compared against biopsy GS (bGS).

**Results:**

All radiomic models outperformed the baseline models. The best-performing (mean ± stdv [%]) single-modality model was the ADC model (76 ± 6%), although not significantly better (p > 0.05) than other single-modality models (T1w: 72 ± 3%, T2w: 73 ± 2%; PET: 75 ± 5%). The overall best-performing model combined PET + ADC radiomics (82 ± 5%). It significantly outperformed most other double-modality (PET + T1w: 74 ± 5%, p = 0.026; PET + T2w: 71 ± 4%, p = 0.003) and single-modality models (PET: p = 0.042; T1w: p = 0.002; T2w: p = 0.003), except the ADC-only model (p = 0.138). In this initial cohort, the PET + ADC model outperformed bGS overall (82.5% vs 72.4%) in the prediction of psGS.

**Conclusion:**

All single- and double-modality models outperformed the baseline models, showing their potential in the prediction of GS, even with an unbalanced cohort. The best-performing model included PET + ADC radiomics, suggesting a complementary value of PSMA-PET and ADC radiomics.

**Supplementary Information:**

The online version contains supplementary material available at 10.1007/s00259-021-05430-z.

## Introduction

Prostate cancer (PCa) is a leading cause of cancer-associated morbidity and mortality in men [1]. Diagnosis of PCa is commonly achieved by ultrasound-guided needle biopsy and can be improved by multiparametric magnetic resonance imaging (mpMRI) [2, 3]. Positron emission tomography (PET) imaging with PCa-specific tracers can help the delineation of suspicious lesions for guiding repeated biopsies or to improve the sensitivity of lesion detection [2, 4]. More recently, ^68^ Ga-radiolabelled prostate-specific membrane antigen PET (PSMA-PET) demonstrated superiority over other imaging modalities and PET radiotracers in localizing primary staging and biochemical recurrent PCa [5–7]. Moreover, [^68^ Ga]Ga-PSMA-11 PET/MRI showed promising results in aiding targeted biopsy after a previous negative biopsy in patients with high suspicion of PCa [8–10].

Patients with histologically confirmed PCa are initially stratified into risk groups according to serum prostate-specific antigen (PSA) levels, histological findings, and digital-rectal examination results [4]. The Gleason score (GS) extracted from biopsy results or after radical prostatectomy (RP) is the main tool for prognosis, and an indicator of the aggressiveness of PCa. Recently, the International Society of Urological Pathology (ISUP) reached a consensus regrouping of the GS into 5 Gleason Grade Groups (GGG) [11], according to their correlation with patient outcome.

However, in approximately one-third of the patients, biopsy GS (bGS) is different from the final GS determined after surgery (postsurgical GS, psGS), with biopsies tending to underestimate cancer aggressiveness [12]. These discrepancies between the two GS can have important implications in patient management. Therefore, accurate determination of PCa aggressiveness by adding pre-therapeutic imaging features is of high clinical interest.

To achieve this goal, data-driven strategies received much interest in the last decade. In this work, we focus on radiomics, which is the extraction of image features from medical images and their use to build models for improved decision support. Hand-crafted radiomic features have been previously applied for aiding detection and prognosis in breast cancer [13], lung cancer [14], and glioma [15]. MRI-only radiomics have also been applied in PCa prognosis, using GS as a proxy [16–18]. PSMA-PET radiomics [19] and other PET tracers [20] have been independently applied in the discrimination between low- to intermediate-risk (GS ≤ 7 or GGG 1–3) and high-risk (GS ≥ 8 or GGG 4–5) PCa.

In this study, we investigated the performance of hand-crafted radiomic features extracted from pre-therapeutic [^68^ Ga]Ga-PSMA-11 PET/MRI in predicting psGS in three categories (GGG 1–3, GGG 4, GGG 5). The selection of the three categories was based on a compromise between a more comprehensive prediction, knowing that all the selected Gleason categories represent different clinical outcomes [21], and the availability of data, since there were not enough patient data to represent every Gleason category.

The complementary value of PET and MRI radiomics was evaluated by comparing single- (PET or MRI) and double-modality (PET + MRI) radiomics. In addition, the performance of image-based models was compared to two baseline models: the first one trained with clinical patient-data only, and the second one trained with volume and maximum intensity radiomic features only. Finally, psGS predictions from the best-performing model were compared to assuming bGS.

## Methods

### Patient population

Patients with histopathologically proven primary adenocarcinoma of the prostate who (i) received a [^68^ Ga]Ga-PSMA-11 PET/MRI at our institution between November 7, 2012, and February 13, 2014, for initial staging of PCa, (ii) had undergone RP, and (iii) had available surgery-obtained GS were included in this retrospective analysis. Out of the 132 screened patients, 101 met the eligibility criteria and were included in this study, whereas 31 patients did not include all the necessary MR images and were excluded. Patient characteristics are summarized in Table 1. Clinical and histopathological information were extracted from hospital database. All patients provided written informed consent for data evaluation and publication. The retrospective data analysis was approved by the medical ethics committee of the Technical University of Munich (reference number: 5665/13S).

**Table. 1:**
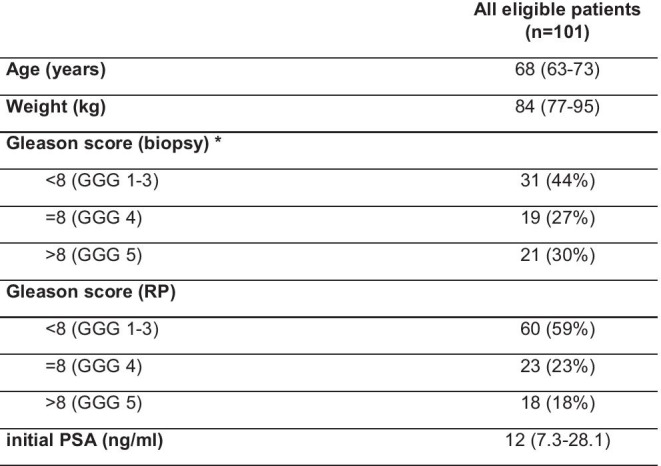
Patient cohort characteristics. Data are median (interquartile range) or n (%); *PSA*, prostate-specific antigen; *RP*, radical prostatectomy; *Missing biopsy results for 30 patients (n = 71)

The histopathology data were extracted from RP pathology reports. GS values were patient-based, meaning that the total GS per patient was selected, consisting of the sum of the scores of the two most dominant Gleason patterns. Patients were grouped by GS into three categories: lower than 8 (GGG 1–3), equal to 8 (GGG 4), and higher than 8 (GGG 5).

### Imaging protocol

Imaging was performed on an integrated whole-body PET/MRI system (Biograph mMR, Siemens Healthineers, Erlangen, Germany) with a 3 T MRI system. PET images were obtained after intravenous injection of a median of 142.0 (interquartile range [IQR]: 118.3–156.8) MBq of [^68^ Ga]Ga-PSMA-11 synthesized as previously described [22]. Twenty milligrams of furosemide were injected right after tracer administration. PET/MRI acquisitions started at a median of 60.4 (IQR: 51.5–73.2) minutes following radiopharmaceutical injection. Subsequently, mpMRI examination of the prostate was performed simultaneously within a 15-min single bed position PET scan (PET), including a coronal T1-weighted image (T1w), an isotropic T2-weighted image (T2w), and an axial apparent diffusion coefficient map (ADC), all centered on the prostate. All acquisition and reconstruction protocols for both PET and MRI sequences were the same for all patients included in the present study (Supplementary Table 1).

### Image segmentation

For the extraction of radiomic features, volumes of interest (VOI) in PSMA-PET and MRI images were first individually segmented. To avoid the limitations of radiomics for small lesions in PET images [23] and the complexities of multi-lesion characterization through hand-crafted radiomic features, whole-prostate segmentations were performed. PSMA-PET images were segmented using a previously validated fuzzy-logically adaptive Bayesian (FLAB) segmentation tool [24], which provides accurate estimation of volumes of interest through modelling of noise and blur characteristics of PET imaging [25]. Whole prostates from MR images (T1w, T2w, and ADC maps) were manually segmented in each modality. The segmentations were performed by a nuclear medicine physician with 3 years of experience in PSMA hybrid imaging.

### Radiomic features extraction

The radiomic features were extracted from the segmented volumes, in accordance to the image biomarker standardization initiative (IBSI) guidelines [26]. Two different discretization approaches were used. To preserve the original intensity scale and meaning of the voxel values, quantitative functional imaging modalities (PSMA-PET and ADC maps) were discretized using fixed bin width (FBW) sizes (bin sizes PET [SUV] = 0.030, 0.060, 0.125, 0.250, 0.500, 1.000; bin sizes ADC [10^−6^ mm^2^/s] = 10, 25, 50, 100, 200, 400). According to the IBSI guidelines, in order to normalize the images and prioritize contrast inside the VOIs, the discretization of non-quantitative MRI T1w and T2w images was performed using fixed bin numbers (FBNs, number of bins = 8, 16, 32, 64, 128, 256) discretization. From these discretization schemes, the best-performing one for each model based on its balanced accuracy was selected as the final model.

**Fig. 1:**
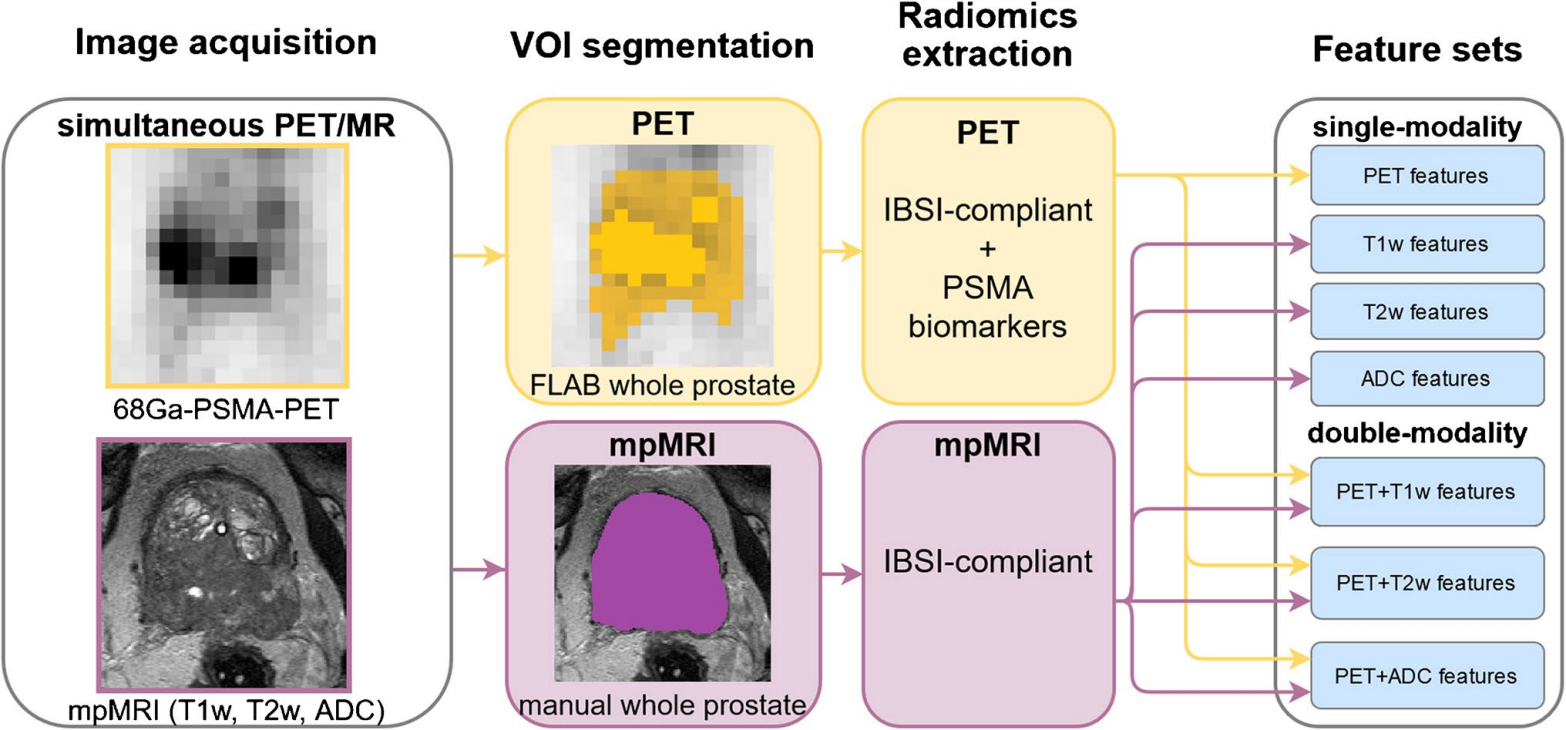
Feature extraction workflow for PSMA-PET and mpMRI images

Overall, 107 3D radiomic features were extracted from the original VOIs without resampling, using PyRadiomics [27], which included: first order (n = 18), shape (n = 14), Gray Level Co-occurrence Matrix (GLCM) (n = 24), Gray Level Size Zone Matrix (GLSZM) (n = 16), Gray Level Run Length Matrix (GLRLM) (n = 16), Neighbouring Gray Tone Difference Matrix (NGTDM) (n = 5), and Gray Level Dependence Matrix (GLDM) (n = 14). The feature extraction workflow is described in Fig. 1.

Based on previous works [28–31], six features derived from commonly used PSMA-PET quantitative biomarkers were also included among the PSMA-PET features: SUV_peak_ (maximum average SUV within a 1-cm^3^ spherical volume), relative SUV_peak_ (ratio of SUV_peak_ and the mean SUV of the VOI), volume of the 40% of SUV_max_ isocontour [40% Volume], volume fraction of the 40% isocontour [40% Fraction], SUV_mean_ in the 40% isocontour [40% SUV_mean_], and “Total SUV” (product of the 40% SUV_mean_ and the 40% Volume). The isocontour volume and the “Total SUV” features were based on “PSMA-ligand tumor volume” [PSMA-TV] and “PSMA-ligand total lesion” [PSMA-TL] [28], adapted to our prostate segmentation.

All the features used in this work are described in the Supplementary Table 2.

### Prediction models

**Fig. 2:**
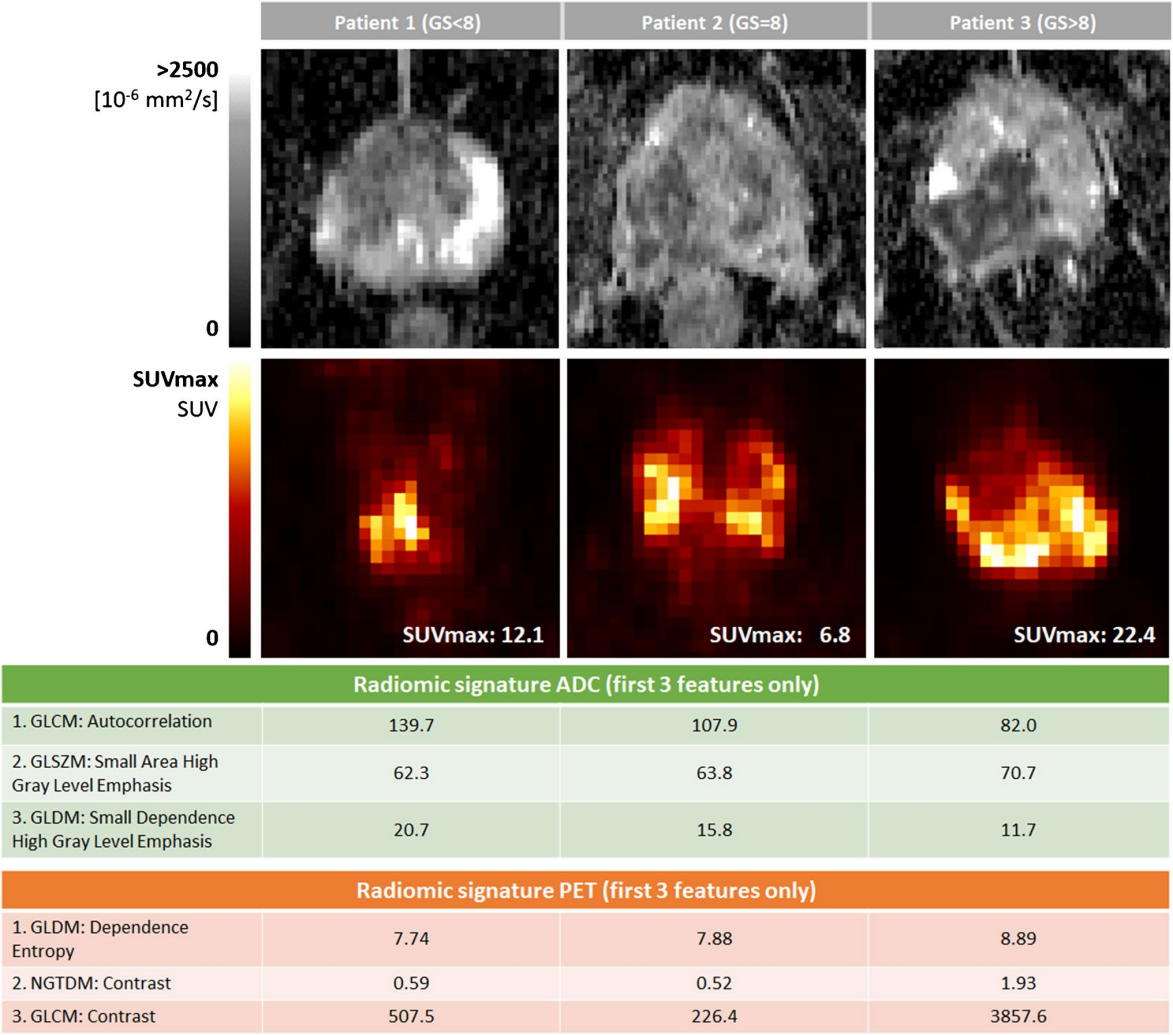
Examples of radiomic signatures (three most relevant features only) of three patients, one per GS category

A machine learning model was trained to identify GS using a 3-class support vector machine (SVM) with a radial basis function (RBF) kernel and a “one-vs-rest” multi-class approach. The SVM was trained with up to 10 previously selected PET and MRI radiomic features, using a recursive feature elimination (RFE) method for the selection of the radiomic signature. The cohort was split into training and validation sets using a sixfold stratified cross-validation, with a 2:1 patient ratio for training (n = 67) and validation (n = 34) in each fold, respectively. As examples of radiomic signatures, the three most relevant features of two models (with ADC and PET radiomics, respectively) are presented in Fig. 2 for three example patients.

**Fig. 3:**
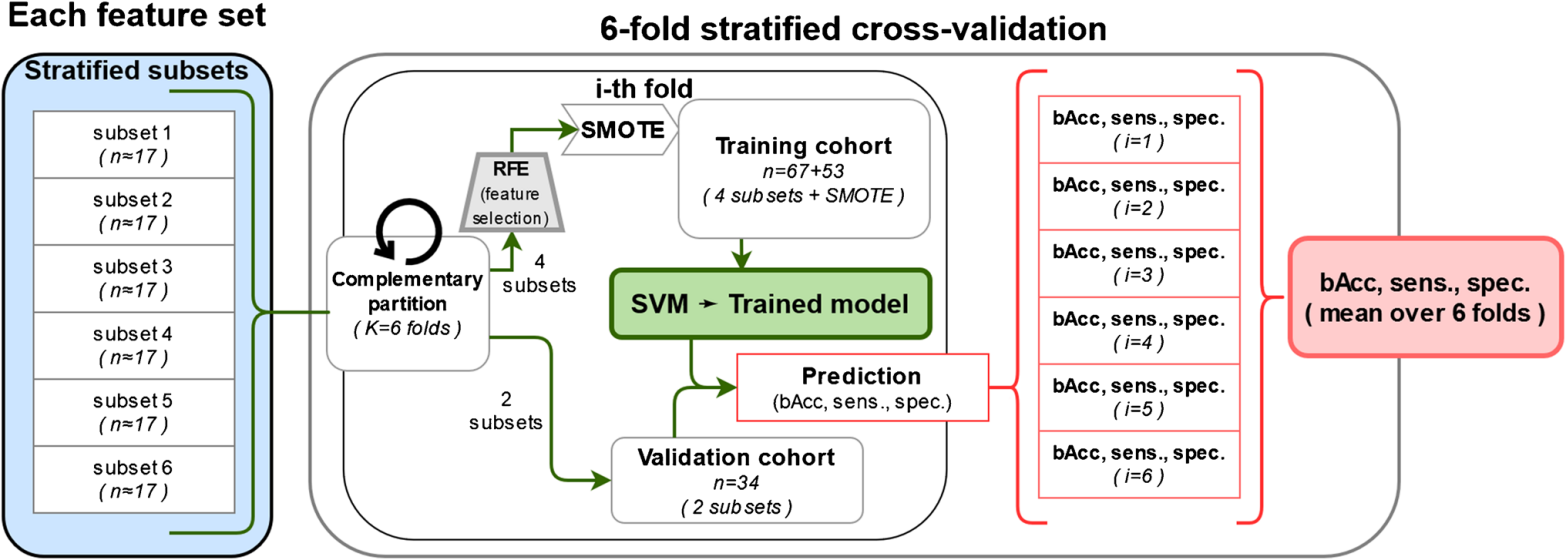
Training and validation workflow of each SVM model for the prediction of GS

Given the strong class imbalance (59% of the patients belong to the class GGG 1–3), a method to balance the training datasets is required. There are several alternatives, which usually imply an oversampling of the less prevalent classes, an undersampling of the most prevalent class, or a combination of both. With a limited dataset, our study is better suited for oversampling techniques, to avoid neglecting important information. The synthetic minority oversampling technique (SMOTE) was applied to oversample all training features in both less prevalent classes (GGG 4 and GGG 5) up to a 1:1 proportion with the most prevalent class (GGG 1–3). The augmented training data was used to train the SVM for each of the 6 cross-validation cycles. The trained models were then tested in the prediction of GS with the non-augmented validation data. The implemented training and validation of the models is displayed in Fig. 3.

Separate models were trained using either radiomic features from a single image type (single-modality models: PSMA-PET, T1w, T2w, ADC) or combined from PET and each MR sequence (double-modality models: PSMA-PET + T1w, PSMA-PET + T2w, PSMA-PET + ADC). The use of more than two image types — such as PET and several MR sequences — was not implemented for two reasons: first, the exponentially higher computing time required to train a model with all the features and combined hyperparameters from many modalities; second, the relative low outcome changes obtained from adding extra modalities in previous experiences [32].

We compared our radiomic models against two ad hoc baseline SVM models to evaluate their performance. With these comparisons, we investigated if our radiomics performed better than using other available information. To study the added value of image radiomics beyond conventional clinical information, a patient-data baseline was established. To confirm that our models did not only rely on surrogates of volume or maximum intensity voxel values, an image-based model (“radiomics baseline”) was trained. This echoes previous studies [23, 33] which suggest that, if left unchecked, some radiomics signatures may be little more than proxies for simpler statistics, like the number of voxels of the VOI (i.e., its volume). Adding such a baseline model helps us discard this hypothesis whenever our models outperform the radiomics baseline, implying that our models rely on more complex features.

For the training of these two baseline models, we followed the same workflow as in the image radiomics models (Fig. 3), but instead of starting with a set of image radiomic features, we used: only relevant patient data (i.e., age, weight, and initial PSA [iPSA]) for the patient-data baseline; only VOI volume and maximum values (i.e., PET VOI volume, SUV_max_, and ADC_max_) for the radiomics baseline.

### Statistical analysis

Since the dataset was unbalanced and classification performance can be different for each of the 3 classes, the results are expressed as the balanced accuracy (bAcc) among the three classes (Eq. 1):

1 $$bAcc=\frac{1}{3}\left(\frac{{TP}_{GGG 1-3}}{{\left(TP+FN\right)}_{GGG 1-3}}+\frac{{TP}_{GGG 4}}{{\left(TP+FN\right)}_{GGG 4}}+\frac{{TP}_{GGG 5}}{{\left(TP+FN\right)}_{GGG 5}}\right);$$

where TP_CLASS_ is the number of True Positive classifications for the class CLASS, and FN_CLASS_ is the number of False Negative classifications for the class CLASS.

The results from the GS predictions are presented as the sixfold mean and standard deviations of bAcc, sensitivities, and specificities of the best prediction using the validation data. Results from the baseline models, PET-only radiomics, each MR-modality (T1w, T2w, ADC) radiomics only, and combined PET + MR radiomics were compared using Student’s t-test (statistical significance: p < 0.05; normality test: Shapiro–Wilk), to evaluate the complementary value of radiomics from both imaging modalities.

To assess the potential clinical value of using PET-MR radiomics in the prediction of GS, we compared the use of biopsy GS (bGS) as a substitute for postsurgical GS against the predictions from our best-performing model. All the predictions from our best-performing model on our validation data were compared to bGS only in the patients with both bGS and psGS available. We inform the balanced accuracy of the predictions, as well as the sensitivity and specificity of the predictions for each GS class.

Since most previous studies saw interest in presenting their results in two categories (lower/intermediate vs high risk), we also present the results of our best-performing model in this fashion (GGG 1–3 vs GGG 4–5).

The statistical analysis was performed using numpy and scipy.stats in Python.

## Results

The 101 patients who met the eligibility criteria were grouped by GS into three categories: GS < 8 (GGG 1–3: 60 [59%] patients), GS equal to 8 (GGG 4: 23 [23%] patients), and GS > 8 (GGG 5: 18 [18%] patients). In each cross-validation cycle, two-thirds of the patients (n = 67) were selected from each class as training data (GGG 1–3: 40, GGG 4: 15, GGG 5: 12), leaving the rest (n = 34) as validation (20, 8, and 6 patients, respectively). The best-performing model (highest average bAcc in the validation) for each image modality was selected for comparison. The characteristics of the selected models are summarized in the Supplementary Table 3.

Three out of four single-modality models (PET, T1w, T2w) used a combination of first order, shape, and textural radiomics features, except the ADC model, which did not include any first order features. All double-modality models (PET + T1w, PET + T2w, PET + ADC) included both PET and MR features.

Across the PET-only model and all double-modality models (all of which include PET radiomics), the most often selected PET features were the shape feature “Maximum 2D Diameter Row” (present in all 4 models) and the quantitative biomarker “Total SUV” (present in 3 out of 4 models).

In both models including ADC features, textural ADC features were predominant over shape and first order features (with ratios 6:1 and 5:2 textural features over the rest, respectively). In both models including T2w features, shape features were the most frequent (4:3 and 5:1, respectively). In the models with T1w features, the selected feature ratio was more evenly distributed among the 3 types (first order/shape/textural: 2:1:6 and 3:1:3, respectively).

**Fig. 4:**
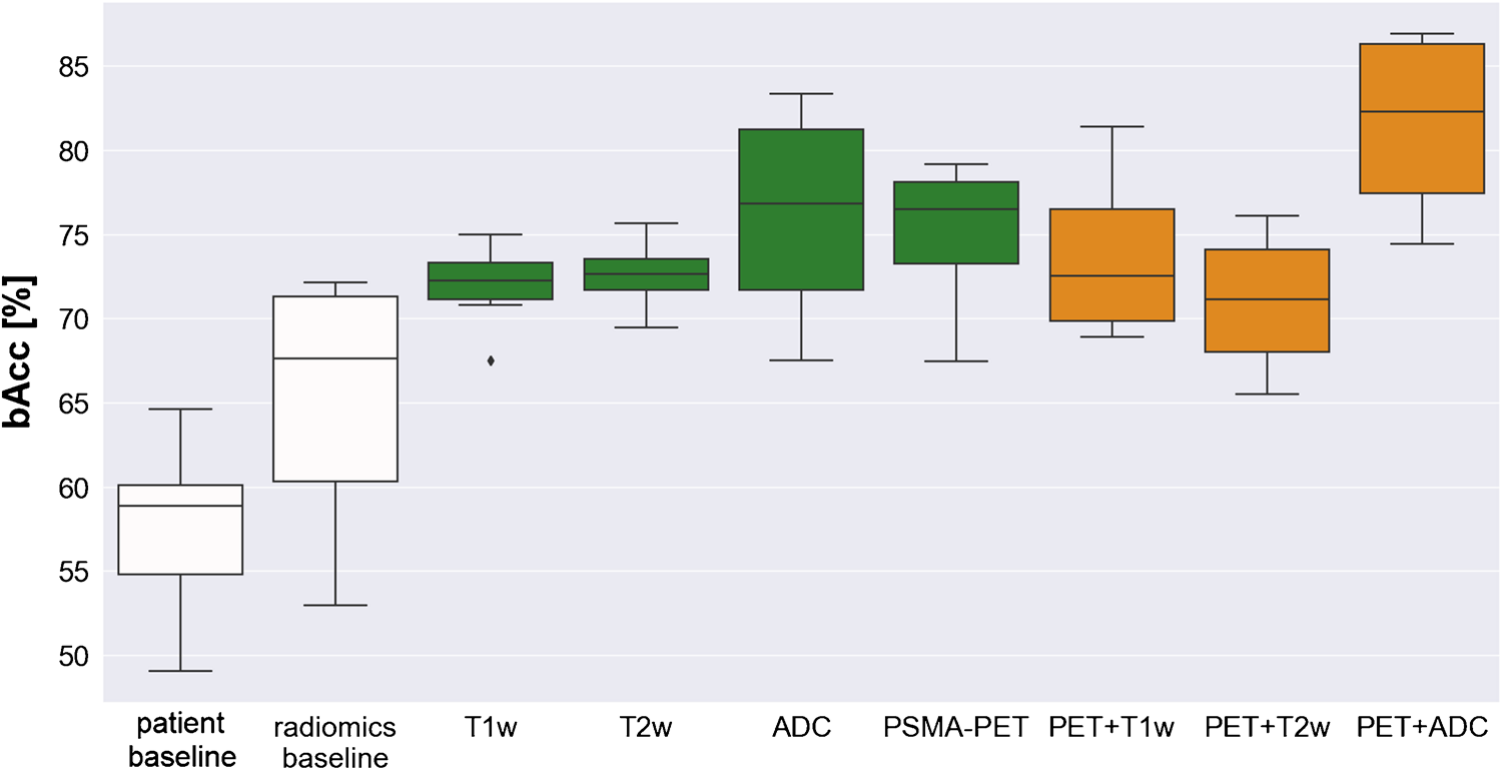
Boxplot of the balanced accuracies of all best-performing models on the validation sets

The performances of the implemented models are summarized in Table 2. For a visual comparison, Fig. 4 displays a box plot of the bAcc of all radiomic and baseline models.

**Table. 2:**
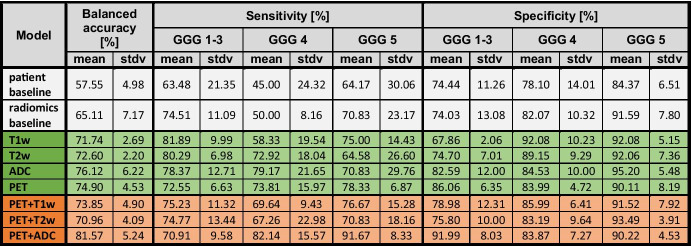
Performances of the trained models (*top*, *grey*: baseline models; *center*, *green*: single-image radiomics; *bottom*, *orange*: double-image radiomics) on the validation dataset, expressed as their balanced accuracies, sensitivities, and specificities (mean and standard deviation, in percentages)

The comparisons between models were performed using the validation bAcc (mean ± standard deviation). For the patient-data baseline model and the radiomics baseline model, the bAcc was 58 ± 5% and 65 ± 7%, respectively. All single-modality models (T1w: 72 ± 3%; T2w: 73 ± 2%; ADC: 76 ± 6%, PET: 75 ± 5%) provided a significantly better classification performance than the patient-data baseline (p < 0.001). The radiomics baseline model, while exhibiting a higher bAcc, did not significantly outperform the patient-data baseline. Most single-modality models outperformed also the radiomics baseline model, except the T1w model (T2w: p = 0.034, ADC: p = 0.018, PET: p = 0.018, T1w model: p = 0.060). Among the single-modality models, the model trained with ADC radiomics provided the highest performance (76 ± 6%), although not statistically higher than any of the other single-modality radiomics models (p > 0.05). The sensitivities of this model were similar across classes, but slightly higher for GGG 5 (sens: GGG 1–3: 73 ± 7%; GGG 4: 74 ± 16%; GGG 5: 78 ± 7).

A model trained with combined features from PSMA-PET and ADC map radiomics yielded the highest overall accuracy (82 ± 5%). It significantly outperformed most single-modality models, including the PET-only (with p = 0.042), T1w-only (p = 0.002), and T2w-only (p = 0.003), while the difference with the best model trained with ADC-only radiomics was not statistically significant (ADC: 76 ± 6%, p = 0.138). The better performance of this model resulted from the highest sensitivity to the higher risk groups but at the cost of a lower sensitivity to the low/intermediate-risk group (sens: GGG 1–3: 71 ± 10%; GGG 4: 82 ± 16%; 92 ± 8%).

The addition of T1w or T2w radiomics to PSMA-PET radiomics (74 ± 5% and 71 ± 4%, respectively) did not significantly alter the performance of single-modality models (p > 0.05). Combined PET + ADC radiomics significantly outperformed both of these double-modality models (PET + ADC: 82 ± 5%; p-value: 0.026 and 0.003, respectively).

P-values for all model combinations are summarized in Table 3.

**Table. 3:**
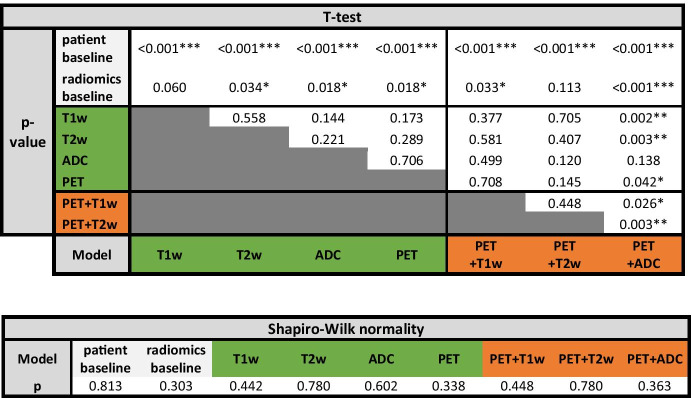
Correlation matrix (p-values) from unpaired t-tests between all model performances (balanced accuracies) and Shapiro–Wilk normality test significance.

Significance levels: *p < 0.05; **p < 0.01; ***p < 0.001; otherwise: not statistically significant (*grey*: baseline models; *green*: single-image models; *orange*: double-image models)

For the best-performing model, the results of the classification of lower/intermediate- vs high-risk Gleason categories (GGG 1–3 vs GGG 4–5) are presented in Table 4. The best-performing baseline model was again the radiomics baseline (74 ± 5%), although not significantly better than the patient baseline (69 ± 8%, p = 0.203). The hybrid PET + ADC model (82 ± 6%) outperformed the patient and radiomics baseline models overall (p = 0.011 and 0.029, respectively), with a much higher sensitivity to GGG 4–5 (patient baseline: 74 ± 11; radiomics baseline: 74 ± 13; PET + ADC: 94% ± 8) at the cost of a slightly poorer sensitivity to GGG 1–3 (patient baseline: 63 ± 21%; radiomics baseline: 75 ± 11%; PET + ADC: 71 ± 10).

**Table. 4:**
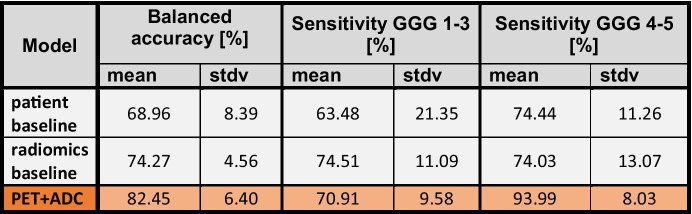
Performances of the baselines vs the best-performing model trained on 3 GS groups (GGG 1–3, GGG 4, GGG 5) and tested on 2 GS groups (GGG 1–3, GGG 4–5) (*top*, *grey*: baseline models; *bottom*, *orange*: double-image radiomics), expressed as their balanced accuracies (mean and standard deviation, in percentages), and sensitivities by class (t-test comparisons: patient baseline vs radiomics baseline: p = 0.203; patient baseline vs PET + ADC: p = 0.011; radiomics baseline vs PET + ADC: 0.029)

From the 71 patients with both available psGS and bGS (GGG 1–3: 62% (n = 44); GGG 4: 20% (n = 14); GGG 5: 18% (n = 13)), our model outperformed the bGS in predicting psGS overall (bAcc: 82.5% vs 72.4%, respectively) and within each group (sensitivity: GGG 1–3: 72.3% vs 68.2%; GGG 4: 84.0% vs 64.3%; GGG 5: 91.3% vs 84.6%, respectively). The comparisons between biopsy GS and the PET + ADC model in predicting psGS can be found in Table 5.

**Table. 5:**
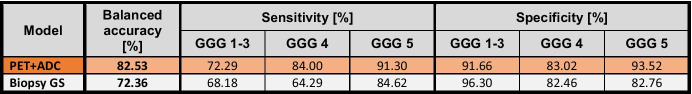
Comparison of the prediction of postsurgical GS (psGS) between our best-performing model (*orange*: PET + ADC radiomics) and the biopsy GS (bGS, *white*) on the patients with both psGS and bGS available, expressed as their balanced accuracies, sensitivities, and specificities (in percentages)

## Discussion

In this work, we studied the potential of hand-crafted radiomics from [^68^ Ga]Ga-PSMA-11 PET/MR images for predicting postsurgical Gleason scores. Previous to this study, the literature on the impact of PET/MRI on PCa radiomics was scarce. In addition, we used a prediction of three GS categories, instead of two as in most previous studies. Important aspects of our work are discussed below: the rationale in choosing our segmentation methods, the results of the feature selection and GS predictions, and limitations and perspectives on future work.

Our segmentation strategy implied a trade-off, which intended to minimize the complexity while achieving effective radiomics. In VOIs with a small number of voxels, radiomics analysis cannot provide much complementary information to the VOI volume [23]. Since PET images have large and rather low-resolution voxels, small prostate lesions are prone to render many radiomic features into surrogates of the number of voxels (i.e., lesion volume). To overcome this limitation, we segmented the whole-prostate gland. Overall, image-based models performed significantly better than the radiomic baseline, indicating that the classification was not merely based on volume surrogates.

Another argument for whole-prostate segmentations was to reduce the complexity of the radiomics workflow. In the case of multi-lesion PCa, a feature extraction process using multiple VOIs would have implied a more complex mechanism to aggregate the radiomics features throughout the lesions. To make matters more complex, PCa lesions are not always simultaneously present in all image modalities, making the segmentations and the feature extraction more cumbersome or even impossible for different images. Our whole-prostate approach ensured not only big enough VOIs (of more than one thousand voxels) but also a simpler segmentation and feature extraction process, with only one VOI per image type. This is also a logical approach from a clinical perspective, since one patient would have different GS values across lesions, but only the highest (index lesion) is considered for treatment and prognosis.

After extracting the features from different VOIs, the weakest features were removed through recursive feature elimination (RFE). RFE works by fitting an SVM model with all the features, and eliminating the feature that the SVM considers less relevant. This process is repeated several times, eliminating one feature at a time, so as to leave only the highest ranked and less interdependent ones. One characteristic of all models was that the prostate radius was always selected as a feature, which may be accounting for the effect of prostate volume in the detection of prostate cancer lesions [17]. In particular, the models containing T2w radiomic features relied mostly on this and other shape features, relegating first order and textural features to a lesser role. These selected radiomic features not only reflect image information that is important for the classification but are also influenced by the chosen segmentation method [31]. The models with ADC features, on the other hand, were the two best-performing models and relied mainly on the textural properties of the prostate, instead of its shape features. Interestingly, most PET models repeatedly selected a commonly used quantitative biomarker based on PSMA-TL, stressing its importance in predicting GS.

The best-performing single-modality models included PSMA-PET (bAcc = 75 ± 5%) and ADC map (bAcc = 76 ± 6%) features, which concordantly are the focus of most previous literature [16–20]. Overall, the best-performing model was the one that combined features from both highest-performing single modalities (PET + ADC, bAcc = 82 ± 5%), demonstrating the added value of the multi-modality approach compared to either PET-only or MR-only radiomics. It is interesting to note that the better performance of this model emerged from a higher sensitivity to high-risk groups (GGG 4 and 5), trading sensitivity in the prediction of the low/intermediate-risk groups (GGG 1–3), which implies a misdiagnose (overgrading) of a considerable percentage of low/intermediate-risk patients. As a trade-off, the specificity of low/intermediate-risk patients and the sensitivity to high-risk patients are greatly improved.

All image-based models performed significantly better than the patient-data baseline, demonstrating that image radiomics provide additional information to the available clinical parameters. Most models also significantly outperformed the radiomics baseline, except for the T1w and PET + T2w models. This implies that, for most models, the selected combinations of features were not mere surrogates of volume or intensity, even though some features that correlate with volume were involved in the classification.

The results from the predictions of low/intermediate- vs high-risk Gleason scores require a special discussion. First, it is important to clarify that, although our model allow the predictions of two categories (by combining the predictions of both upper categories, GGG 4 and GGG 5), it was trained for the prediction of three categories. Since it was not optimally trained for this prediction, it would most likely underperform in comparison to a real two-category model. Second, we trained our model to optimize the balanced accuracy, which has no bias towards a particular category. In combining two categories, we are imposing a bias towards these categories, hindering the remaining category. Even so, the overall predictions of the PET + ADC model (82 ± 6%) were still superior to both baseline models (69 ± 8% and 74 ± 5%), at the cost of a lower sensitivity to the low/intermediate-risk category.

As we mentioned, our study has potential clinical implications. Given that not every patient undergoes radical prostatectomy, the biopsy GS is usually used instead of the postsurgical GS in the clinical routine, even though they are not always equivalent. Our hybrid radiomics model outperformed the use of biopsy GS in estimating the postsurgical GS (82.5% vs 72.4%). It is important to note that our dataset lacked biopsy GS for around 30% of the patients. With a larger initial cohort (implying more patients with simultaneous postsurgical and biopsy GS data), we would be able to analyze the power of combined biopsy GS and image radiomics in the prediction of postsurgical GS. As an alternative, we could also use the biopsy GS as part of the patient-data baseline, as a more clinically relevant baseline model.

Our study is not without limitations. Firstly, familywise error rates (FEWRs) across the statistical analyses were not controlled, meaning that several comparisons between models were performed through a statistical test (i.e., Student’s t-test), without correcting for the higher probability of Type I errors. Secondly, our results show high intragroup variances in the performance of most classifiers (Table 2). In particular, even though the model trained with PET + ADC radiomics outperformed the rest of the models in terms of balanced accuracy, the difference with respect to the ADC-only model was not statistically significant. The high variances can be partly attributed to the small number of patients and strongly unbalanced dataset, which implies that, in each fold, only radiomics from around 15 or 12 patients (GGG 4 or GGG 5, respectively) were used for training the models, and 8 or 6 patients (GGG 4 or GGG 5, respectively) were used for evaluation in the less prevalent GS classes. More robust models with lower variability between successive training cycles would require a larger cohort and, ideally [34], an external testing cohort.

Although GS is widely used as a proxy for the aggressiveness of PCa, it must be used with caution. Studies show that, in some cases, the fraction of Gleason patterns relate to the outcome of patients better than the GS [35]. For instance, GS 7 tumors represent a rather diverse population and, as a consequence, there is clinical value in further differentiating it in subcategories. In our work, we focused on demonstrating that [^68^ Ga]Ga-PSMA-11 PET and mpMRI would synergically work in the prediction of GS, but our results could profit from considering the outcome of the patients beyond GS.

In our work, we took advantage of the benefits of whole-prostate segmentations, but our segmentation approach has room for improvement. The PI-RADS v2 protocol [36] proposes a two-region approach for PCa diagnosis with mpMRI. According to the protocol, DWI/ADC map is the most informative sequence for the assessment of PCa in the peripheral zone (PZ), while T2w is used mainly for assessment of PCa in the transition zone (TZ) of the prostate. To implement this, PZ/TZ segmentation would mean a more demanding segmentation work for the radiologists, although, for big cohorts, it could also be automated by appropriately training a deep learning (DL)-based algorithm.

DL techniques can also be exploited for feature extraction and prediction. Our decision of using hand-crafted radiomics was based on their higher level of interpretability, as well as on the existence of several previous studies. On the contrary, DL features are more complex to decipher, requiring specific methodologies to “open the black box” and provide an explanation of the output classification. One such methodology is the use of activation or saliency maps of attention, and their relation to correct and incorrect classifications [37, 38]. Another applicable approach is training a fully convolutional network for segmentation of a pathological tissue (e.g., a tumor), and using its trained semantic layers as features for the classification of the pathology [39]. An explainable DL approach could help save time and impact the performance of our predictions, especially in the case of bigger patient cohorts. There are already several works predicting GS from mpMRI-only radiomics using non-explainable deep convolutional layers [40–42]. Based on our results and available technology, a step forward would benefit from including also PSMA-PET radiomics and some form of explainable DL approach.

## Conclusion

Our work shows promising results on the combined power of PSMA-PET and mpMRI radiomic features for predicting postsurgical GS in PCa patients and envisions a reliable tool that helps urologists and radiologists in their daily decision-making process.

## Supplementary Information

Below is the link to the electronic supplementary material.

Supplementary file1 (PDF 287 KB)

Supplementary file2 (PDF 291 KB)

Supplementary file3 (PDF 158 KB)

## Data Availability

The datasets generated and/or analyzed during the current study are available from the corresponding author on reasonable request.

## Abbreviations

ADC: Apparent diffusion coefficient
bAcc: Balanced accuracy
bGS: Biopsy Gleason score
DWI: Diffusion weighted image
FLAB: Fuzzy-logically adaptive Bayesian
GGG: ISUP Gleason Grade Groups
GS: Gleason score
IBSI: Image biomarker standardization initiative
IQR: Interquartile range
ISUP: International Society of Urological Pathology
mpMRI: Multiparametric MRI
MRI: Magnetic resonance imaging
PCa: Prostate cancer
PET: Positron emission tomography
PSA: Prostate-specific antigen
psGS: Postsurgical Gleason score
PSMA: Prostate-specific membrane antigen
RBF: Radial basis function
RFE: Recursive feature elimination
RP: Radical prostatectomy
stdv: Standard deviation
SMOTE: Synthetic minority oversampling technique
SUV: Standard uptake value
SVM: Support vector machine
T1w: T1-weighted MR image
T2w: T2-weighted MR image
VOI: Volume of interest

## Code availability

Questions regarding the code implemented in this work can be addressed to the corresponding author.
