# Supplementary material for: The added value of PSMA PET/MR radiomics for prostate cancer staging
Source: Eur J Nucl Med Mol Imaging. 2021 Jul 13;49(2):527–38. doi: 10.1007/s00259-021-05430-z (PMC8803696; doi:10.1007/s00259-021-05430-z)
Supplement: Supplementary file 1 — Supplementary file1 (PDF 287 KB) [file 259_2021_5430_MOESM1_ESM.pdf]

Article title: The added value of PSMA PET/MR radiomics for prostate cancer staging
Journal: European Journal of Nuclear Medicine and Molecular Imaging
Authors: Solari EL, Gafita A, Schachoff S, Bogdanović B, Villagrán Asiares A, Amiel T, Hui W, Rauscher I, Visvikis D, Maurer T, Schwamborn K, Mustafa M, Weber W, Navab N, Eiber M, Hatt M, Nekolla SG

	**PET**	**T1w**	**T2w**	**ADC map**
**Image description**	^{68}Ga-PSMA-11 image (with MLAA-based attenuation correction OP-OSEM reconstruction)	T1 weighted FSE (TR: 600, TE: 8.7, ETL: 5)	T2 weighted SPACE (TR: 1700, TE: 102, ETL: 89)	ADC map (b-values: 50, 400, 800)
**Rows x Columns**	172x172	264x384	320x320	136x160
**Pixel size [mm]**	4.17 / 4.17	1.17 / 1.17	0.72 / 0.72	1.63 / 1.63
**Slices**	418-516	40	72	20
**Slice thickness [mm]**	2.03	6.50	1.00	3.60
**Corrections**	detector normalization, dead time, attenuation through MLAA, 3D scatter (model based), decay, delayed event subtraction (randoms)			
**Filter**	4mm Gauss XYZ			

Supplementary Table 1. Image characteristics of the acquired PET and MR studies.

Corresponding author: Esteban Lucas Solari. Technical University Munich, School of Medicine, Department of Nuclear Medicine, Munich, Germany.
Contact: elucas.solari@tum.de
