# Supplementary material for: The added value of PSMA PET/MR radiomics for prostate cancer staging
Source: Eur J Nucl Med Mol Imaging. 2021 Jul 13;49(2):527–38. doi: 10.1007/s00259-021-05430-z (PMC8803696; doi:10.1007/s00259-021-05430-z)
Supplement: Supplementary file 2 — Supplementary file2 (PDF 291 KB) [file 259_2021_5430_MOESM2_ESM.pdf]

Article title: The added value of PSMA PET/MR radiomics for prostate cancer staging
Journal: European Journal of Nuclear Medicine and Molecular Imaging
Authors: Solari EL, Gafita A, Schachoff S, Bogdanović B, Villagrán Asiares A, Amiel T, Hui W, Rauscher I, Visvikis D, Maurer T, Schwamborn K, Mustafa M, Weber W, Navab N, Eiber M, Hatt M, Nekolla SG

Feature type	Features
**patient baseline (n=3)**	Age, Weight, Initial PSA
**radiomics baseline (n=3)**	First order, PET: Maximum; Shape, PET: Volume; First order, ADC: Maximum
PET extra features (n=6)	Peak SUV, Relative Peak SUV, 40% Volume, 40% Volume Fraction, 40% Mean SUV, Total SUV
First order (n=18)	10th Percentile, 90th Percentile, Energy, Intensity Histogram Entropy, Interquartile Range, Kurtosis, Maximum, Mean Absolute Deviation, Mean, Median, Minimum, Range, Robust Mean Absolute Deviation, Root Mean Squared, Skewness, Total Energy, Intensity Histogram Uniformity, Variance
Shape (n=14)	Elongation, Flatness, Least Axis Length, Major Axis Length, Maximum 2D Diameter Column, Maximum 2D Diameter Row, Maximum 2D Diameter Slice, Maximum 3D Diameter, Volume, Minor Axis Length, Sphericity, Surface Area, Surface Volume Ratio, Approximate Volume
GLCM (n=24)	Autocorrelation, Cluster Prominence, Cluster Shade, Cluster Tendency, Contrast, Correlation, Difference Average, Difference Entropy, Difference Variance, Id, Idm, Idmn, Idn, Imc1, Imc2, Inverse Variance, Joint Average, Angular Second Moment, Joint Entropy, MCC, Joint maximum, Sum Average, Sum Entropy, Joint Variance
GLSZM (n=16)	Gray Level Non Uniformity, Gray Level Non Uniformity Normalized, Gray Level Variance, High Gray Level Zone Emphasis, Large Area Emphasis, Large Area High Gray Level Emphasis, Large Area Low Gray Level Emphasis, Low Gray Level Zone Emphasis, Size Zone Non Uniformity, Size Zone Non Uniformity Normalized, Small Area Emphasis, Small Area High Gray Level Emphasis, Small Area Low Gray Level Emphasis, Zone Entropy, Zone Percentage, Zone Variance
GLRLM (n=16)	Gray Level Non Uniformity, Gray Level Non Uniformity Normalized, Gray Level Variance, High Gray Level Run Emphasis, Long Run Emphasis, Long Run High Gray Level Emphasis, Long Run Low Gray Level Emphasis, Low Gray Level Run Emphasis, Run Entropy, Run Length Non Uniformity, Run Length Non Uniformity Normalized, Run Percentage, Run Variance, Short Run Emphasis, Short Run High Gray Level Emphasis, Short Run Low Gray Level Emphasis
NGTDM (n=5)	Busyness, Coarseness, Complexity, Contrast, Strength
GLDM (n=14)	Dependence Entropy, Dependence Non Uniformity, Dependence Non Uniformity Normalized, Dependence Variance, Gray Level Non Uniformity, Gray Level Variance, High Gray Level Emphasis, Large Dependence Emphasis, Large Dependence High Gray Level Emphasis, Large Dependence Low Gray Level Emphasis, Low Gray Level Emphasis, Small Dependence Emphasis, Small Dependence High Gray Level Emphasis, Small Dependence Low Gray Level Emphasis

Supplementary Table 2. List of all extracted radiomic features

Corresponding author: Esteban Lucas Solari. Technical University Munich, School of Medicine, Department of Nuclear Medicine, Munich, Germany.
Contact: elucas.solari@tum.de
