# Supplementary material for: The added value of PSMA PET/MR radiomics for prostate cancer staging
Source: Eur J Nucl Med Mol Imaging. 2021 Jul 13;49(2):527–38. doi: 10.1007/s00259-021-05430-z (PMC8803696; doi:10.1007/s00259-021-05430-z)
Supplement: Supplementary file 3 — Supplementary file3 (PDF 158 KB) [file 259_2021_5430_MOESM3_ESM.pdf]

**Article title: The added value of PSMA PET/MR radiomics for prostate cancer staging**
**Journal**: European Journal of Nuclear Medicine and Molecular Imaging
**Authors**: Solari EL, Gafita A, Schachoff S, Bogdanović B, Villagrán Asiares A, Amiel T, Hui W, Rauscher I, Visvikis D, Maurer T, Schwamborn K, Mustafa M, Weber W, Navab N, Eiber M, Hatt M, Nekolla SG

| Model | Discretization | Selected features |
|---|---|---|
| **patient baseline** | - | **n=3**: age, weight, iPSA |
| **radiomics baseline** | - | **n=3:** PET VOI volume, PET SUV max, ADC max |
| **T1w** | 32 bins | **n=9**: 1. Skewness; 2. GLDM: Large Dependence High Gray Level Emphasis; 3. Minimum; 4. Max 2D Diameter Column; 5. GLSZM: Zone Variance; 6. GLSZM: High Gray Level Zone Emphasis; 7. NGTDM: Complexity; 8. GLSZM: Gray Level Variance; 9. GLCM: Cluster Tendency |
| **T2w** | 8 bins | **n=7**: 1. Minor Axis Length; 2. Maximum 2D Diameter Column; 3. GLRLM: Gray Level Non Uniformity; 4. Surface Area; 5. Least Axis Length; 6. Kurtosis; 7. GLSZM: Size Zone Non Uniformity |
| **ADC** | bins of 200 [$10^{-6}$ $mm^2/s$] | **n=7**: 1. GLCM: Autocorrelation; 2. GLSZM: Small Area High Gray Level Emphasis; 3. GLDM: Small Dependence High Gray Level Emphasis; 4. GLDM: Dependence Variance; 5. GLCM: Joint Entropy; 6. GLSZM: Large Area Low Gray Level Emphasis; 7. Maximum 2D Diameter Column |
| **PET** | bins of 0.25 SUV | **n=9**: 1. GLDM: Dependence Entropy; 2. NGTDM: Contrast; 3. GLCM: Contrast; 4. NGTDM: Complexity; 5. 40% mean SUV; 6. Maximum 2D Diameter Row; 7. 90th Percentile; 8. GLDM: High Gray Level Emphasis; 9. Total SUV |
| **PET+T1w** | PET: bins of 0.25 SUV<br><br>T1w: 32 bins | **n=10**: 1. PET: Maximum 2D Diameter Row; 2. T1w: Minimum 3. T1w: Maximum 2D Diameter Column; 4. T1w GLDM: Dependence Variance; 5. T1w GLSZM: Large Area Low Gray Level Emphasis; 6. T1w GLSZM: Zone Variance; 7. PET GLDM: Dependence Entropy; 8. PET: Total SUV; 9. T1w: Kurtosis; 10. T1w: $90^{th}$ Percentile |
| **PET+T2w** | PET: bins of 1 SUV<br><br>T2w: 16 bins | **n=7**: 1. T2w: Minor Axis Length; 2. T2w: Mesh Volume; 3. T2w: Maximum 2D Diameter Column; 4. PET: Maximum 2D Diameter Row; 5: T2w: Surface Area; 6: T2w GLDM: Dependence Non Uniformity; 7. T2w: Least Axis Length |
| **PET+ADC** | PET: bins of 0.25 SUV<br><br>ADC: bins of 200 [$10^{-6}$ $mm^2/s$] | **n=9**: 1. PET: Maximum 2D Diameter Row; 2. ADC GLDM: Small Dependence High Gray Level Emphasis; 3. ADC GLDM: Dependence Variance; 4. ADC: Maximum 2D Diameter Column; 5. ADC: Surface Area; 6. ADC GLSZM: Large Area High Gray Level Emphasis; 7. ADC GLSZM: Large Area Emphasis; 8: ADC GLRLM: Gray Level Variance; 9. PET: Total SUV |

**Supplementary Table 3.** Characteristics of the best-performing models for each image modality (*top, grey*: baseline models; *center, green*: single-image radiomics; *bottom, orange*: double-image radiomics). The “baseline” models were trained with clinical data only and common radiomics only, respectively.

**Corresponding author: Esteban Lucas Solari**. Technical University Munich, School of Medicine, Department of Nuclear Medicine, Munich, Germany.
Contact: elucas.solari@tum.de
